# Clinical Utility of Trio Exome Sequencing in Rwandan Children With Autism Spectrum Disorder

**DOI:** 10.1002/mgg3.70294

**Published:** 2026-09-15

**Authors:** Olivier Hakizimana, Janvier Hitayezu, Jeanne P. Uyisenga, Norbert Dukuze, Marie Viviane Akimana, Laurence Mizero, Claudine Bampire, Charles Mudenge, Xavier Kanyambari Butoto, Laura Helou, Benoit Charloteaux, Jean‐Hubert Caberg, Vinciane Dideberg, Leonor Palmeira, Abdullateef Isiaka Alagbonsi, Vincent Bours, Annette Uwineza

**Affiliations:** ^1^ Postgraduate Studies, School of Health Sciences, College of Medicine and Health Sciences University of Rwanda Kigali Rwanda; ^2^ Department of Biochemistry, Molecular Biology and Genetics, School of Medicine and Pharmacy, College of Medicine and Health Sciences University of Rwanda Kigali Rwanda; ^3^ Center for Human Genetics, Centre Hospitalier Universitaire Sart‐Tilman University of Liege Liege Belgium; ^4^ Department of Pediatrics University Teaching Hospital of Kigali (CHUK) Kigali Rwanda; ^5^ Department of Biology, College of Science and Technology University of Rwanda Kigali Rwanda; ^6^ Department of Psychiatry Ndera Neuropsychiatric Teaching Hospital Kigali Rwanda; ^7^ Department of Psychiatry and Behavioral Sciences, School of Medicine and Pharmacy, College of Medicine and Health Sciences University of Rwanda Kigali Rwanda; ^8^ Department of Physiology, School of Medicine and Pharmacy, College of Medicine and Health Sciences University of Rwanda Kigali Rwanda

**Keywords:** autism spectrum disorder, copy number variation, Rwandan population, single nucleotide variation, whole‐exome sequencing

## Abstract

**Introduction:**

Autism spectrum disorder (ASD) is a neurodevelopmental condition with substantial genetic and phenotypic heterogeneity. However, populations of African ancestry remain underrepresented in genomic studies, limiting understanding of ASD genetic architecture. This study aimed to characterize rare, clinically relevant genetic variants in a Rwandan pediatric ASD cohort using trio‐based whole‐exome sequencing (WES).

**Methods:**

Trio‐based WES was performed in 31 Rwandan pediatric patients with ASD (aged 2–18 years) and their parents. Variants were analyzed using a trio‐based workflow and classified according to American College of Medical Genetics and Genomics/Association for Molecular Pathology (ACMG/AMP) guidelines.

**Results:**

Eleven candidate variants were identified in 9 of 31 patients, including four likely pathogenic variants and seven variants of uncertain significance. This resulted in a diagnostic yield of 12.9% (4/31), expanded to 29.0% when phenotypically concordant variants of uncertain significance were considered. Most likely pathogenic variants were identified in individuals with syndromic ASD who presented with intellectual disability, epilepsy, and global developmental delay. Likely pathogenic findings included two single nucleotide variants in *GABRB3*, *SYNGAP1*, and two copy‐number variants involving the *GNAS* locus and chromosome 1p35.3‐p35.2.

**Conclusions:**

The diagnostic yield observed in this cohort is consistent with previous trio‐based WES studies of ASD. The findings support the clinical utility of WES for the genetic evaluation of ASD and underscore the need for expanded genomic studies in African populations.

## Graphical abstract

Addressing the Gap: First trio‐based WES study of ASD in Rwanda, addressing African underrepresentation in global genomics. Diagnostic Yield: 12.9% yield of clinically relevant variants, consistent with global benchmarks (10%–30%). Genetic Findings: Confirmed *de novo* likely pathogenic variants identified in high‐impact ASD genes, including *SYNGAP1* and *GABRB3*. Phenotypic Association: Significant enrichment of variant identification in syndromic ASD cases involving epilepsy and global developmental delay. Global Impact: The inclusion of African genomic data enhances global variant interpretation and advances diagnostic equity.

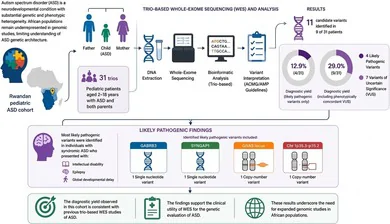

## 1 Introduction

Autism Spectrum Disorder (ASD) is a heterogeneous neurodevelopmental condition characterized by restricted and repetitive patterns of behavior, interests, or activities as well as deficiencies in social communication (Mottron and Bzdok 2020). The diagnosis and treatment of ASD, which often appears early in development, can be made more difficult by comorbid conditions like intellectual disability (ID), seizures, and attention‐deficit/hyperactivity disorder (Lord et al. 2020). ASD is a worldwide public health issue that affects roughly 1 in 100 children (Zeidan et al. 2022). However, estimates of the prevalence of ASD in African populations are inconsistent (Seif Eldin et al. 2008; Lagunju et al. 2014).

Significant progress in genomic research in recent years has demonstrated that ASD has a strong genetic foundation, with heritability estimates ranging from 60% to 90% (Bai et al. 2019). Recent findings from extensive genome‐wide association studies (GWAS), exome sequencing, and copy number variant (CNV) research suggest that the pathophysiology of ASD is influenced by both common polygenic risk factors and rare high‐impact variants (Grove et al. 2019; Satterstrom et al. 2020). However, ASD in the African populations remains understudied at the genomic level, and most genetic association studies have been limited by small sample sizes and lack of representation (Hakizimana et al. 2024).

Whole‐exome sequencing (WES) has proven especially useful in identifying rare pathogenic variants in individuals with ASD, particularly in those with syndromic cases, where ASD is accompanied by additional medical or developmental abnormalities (Fu et al. 2022; Palasantzas et al. 2023).

Current clinical guidelines recommend testing for Fragile X syndrome as a first‐tier genetic test in individuals with ASD prior to the use of broader genomic approaches such as WES, given its status as the most common inherited cause of intellectual disability and a well‐established monogenic contributor to ASD (Hyman et al. 2020).

Syndromic ASD accounts for a substantial proportion of the severe and medically complex cases of ASD. High‐impact variants in genes involved in synaptic signaling pathways, synapse formation, and chromatin remodeling are frequently implicated in these cases (Rylaarsdam and Guemez‐Gamboa 2019). However, a significant proportion of syndromic ASD cases remain genetically unexplained despite advances in genomic technologies.

To address the gaps in the representation of genetic variation from African populations, we conducted a trio‐based WES in a cohort of Rwandan pediatric patients with ASD. Trio‐based WES enabled identification of both inherited and *de novo* variants associated with ASD risk. Increasing representation of historically underrepresented populations is essential to ensure that advances in ASD genomics are globally applicable. By focusing on a Rwandan cohort, this study aimed to identify ASD‐associated variants that may contribute to the genetic architecture of ASD in this population.

## 2 Methods

### 2.1 Study Design and Site

This retrospective, hospital‐based genetic study was conducted at Ndera Neuropsychiatric Teaching Hospital (NNPTH), a national tertiary referral and teaching hospital located in Kigali, Rwanda. NNPTH provides specialized care for neuropsychiatric and neurodevelopmental disorders and receives referrals nationwide.

### 2.2 Study Population and Sampling Procedure

**FIGURE 1:**
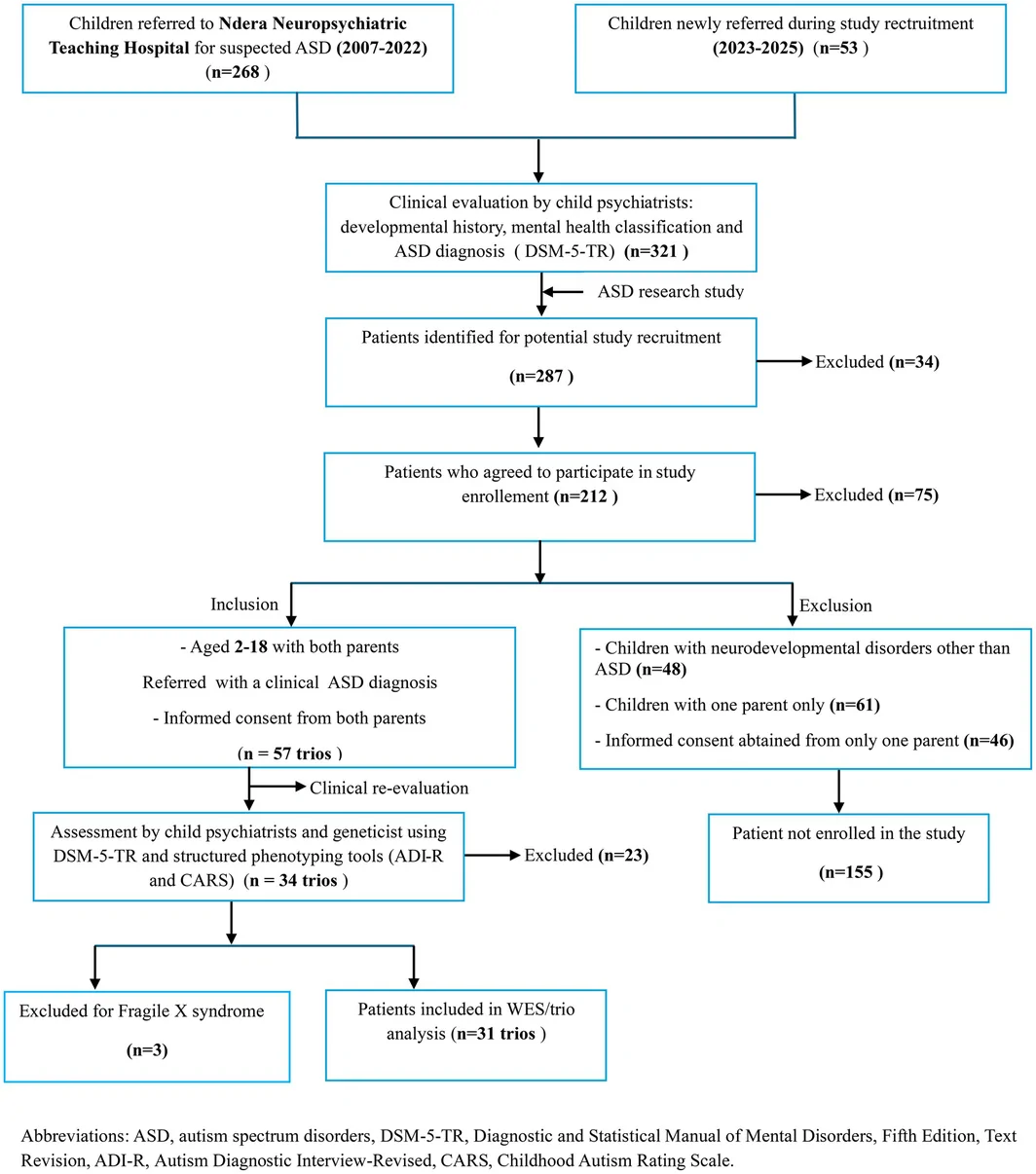
Flowchart of patient recruitment and enrollment for the ASD study.

Patients referred and diagnosed with ASD between 2007 and 2025 (age range of 2 to 18 years) were identified through the NNPTH clinical database and subsequently contacted and invited to participate in the study. ASD was diagnosed clinically based on the Diagnostic and Statistical Manual of Mental Disorders, Fifth Edition, Text Revision (DSM‐5‐TR) (Roehr 2013), and the Autism Diagnostic Interview‐Revised (ADI‐R) (Zander et al. 2017) tools. The severity of autism symptoms was evaluated using the Childhood Autism Rating Scale (CARS) (Schopler et al. 2010). Each participant underwent a comprehensive evaluation, which included: (1) a detailed clinical history, including demographic data, past medical and family history, (2) a general examination to identify any additional dysmorphic features and congenital malformations, and (3) confirmation of the diagnosis of ASD. Selected patients were classified as syndromic ASD and non‐syndromic ASD. Syndromic ASD was defined as ASD accompanied by ID and/or additional congenital anomalies or neurological features including global developmental delay, epilepsy, dysmorphic features, or congenital anomalies. Non‐syndromic ASD was defined as isolated ASD without these associated syndromic manifestations. The patient recruitment process is summarized in Figure 1. Written and signed informed consent for participation was obtained from parents. Only families with available trio samples (affected children and both biological parents) were included.

The sample size was not determined a priori. This retrospective study included all available and eligible ASD trios recruited during the study period at Ndera Neuropsychiatric Teaching Hospital. The sample size was constrained by participant availability and sequencing resources, and the study was designed as an exploratory analysis.

### 2.3 DNA Sample Preparation

DNA samples from proband and both parents were used for genetic analysis. Genomic DNA from the probands and parents was isolated from peripheral blood samples using QIAamp Blood Kit (Qiagen, Germany) at Kigali University Teaching Hospital (CHUK) laboratory, Rwanda. Thereafter, DNA was shipped to the Center of Human Genetics at the University of Liege, Belgium, for WES analysis.

### 2.4 DNA Quantification and Library Preparation

Genomic DNA was quantified using the Qubit 4.0 Fluorometer and normalized to the lowest concentration among the samples within each trio. A volume of 30 μL of normalized DNA was used for library preparation according to the manufacturer's protocol.

Library preparation was performed manually in a diagnostic‐grade pre‐PCR environment using a Veriti Thermal Cycler and standard molecular biology equipment. Tagmentation was carried out using a bead‐based transposome complex, simultaneously fragmenting and tagging genomic DNA with adapters as per the manufacturer's “Illumina DNA Prep with Enrichment” protocol. This step generates normalized libraries with a tight fragment size distribution from a wide DNA input range. After limited‐cycle PCR, libraries were pooled, denatured, and hybridized with Illumina Exome Panel v1.2 probes. Targeted‐enriched fragments were captured using Streptavidin Magnetic Beads (SMB), eluted, amplified, and quantified by Qubit 4.0 Fluorometer prior to whole‐exome sequencing.

### 2.5 Whole Exome Sequencing and Bioinformatics Analysis

Trio‐based whole‐exome sequencing (WES) was performed on 31 ASD families at an accredited clinical genetics laboratory of the University Teaching Hospital of Liège, Belgium, operating under validated protocols and stringent quality control procedures. Exome sequencing was performed using the sequencing‐by‐synthesis technology on the NovaSeq6000 platform (paired‐end; 2 × 100 bp reads; Illumina San Diego, CA, USA) following exome enrichment with the Twist Human Core Exome kit supplemented with additional probes targeting human RefSeq transcripts (ref 102032, Twist Bioscience, South San Francisco, CA, USA). Sequencing achieved a median coverage depth of approximately 200×, with more than 97% of targeted regions covered at a depth greater than 30×, ensuring high confidence in variant detection. Single nucleotide variants (SNVs) and indels as well as copy number variants (CNVs) were respectively called and annotated using homemade bioinformatic pipelines Humanomics (Harvengt et al. 2023) (for SNPs and indels) and StueckFinder (for CNVs).

The laboratory is accredited according to ISO 15189 (BELAC) standards. The Humanomics and StueckFinder pipelines were clinically validated using Genome in a Bottle (GIAB) HG001 and HG002 reference genomes. All reported variants were located within validated high‐confidence genomic regions; therefore, orthogonal confirmation by Sanger sequencing was not required according to the laboratory's validation protocol. Trio relatedness was confirmed using Somalier prior to downstream analyses.

Sequencing reads were aligned to the reference genome (GRCh37/HG19) using Burrows‐Wheeler Aligner (BWA) (Poplin et al. 2018), and variant calling was performed following the GATK Best Practices workflow (Venselaar et al. 2010), using the laboratory's clinically validated diagnostic pipeline. Picard tools (v2.5.0, https://broadinstitute.github.io/picard/) were used to remove duplicate reads. All genomic variants, including single nucleotide variants (SNVs) and Insertions/Deletions (indels), were detected and filtered by GATK Haplotype Caller (Broad Institute, Cambridge, MA, USA). In addition to SNVs and small indels, copy number variants (CNVs) analysis was conducted from BAM files (BWA v0.7.17, elprep v4.1.5) using the Humanomics BED file and a normalization cohort of 400 individuals. CNVs spanning one or more exons were identified using the in‐house StueckFinder v2.2 pipeline, which integrates CANOES with adaptations for gonosomal CNV detection (Backenroth et al. 2014). Candidate CNVs were primarily screened for population frequency and known disease associations using publicly available databases, including gnomAD (Karczewski et al. 2020), the Database of Genomic Variants (DGV) (MacDonald et al. 2014), DECIPHER (Firth et al. 2009), and OMIM (Hamosh 2004). CNV pathogenicity was classified in accordance with the ACMG and ClinGen technical standards (Richards et al. 2015). The SaaS platform Alissa Interpret (Agilent, Santa Clara, CA, USA) was used for variant filtering and annotation (SNVs, indels and CNVs). Data analysis focused on locally curated neurodevelopmental disorder (NDD) gene panel comprising 233 genes associated with ASD and neurodevelopmental disorders (gene list available upon request).

### 2.6 Filtering and Variant Prioritization

All variants were screened based on population allele frequency, genomic location, variant type, published literature, and evidence from public variant databases (ClinVar database, HGMD). Variants with minor allele frequency ((MAF) ≥ 0.01) were excluded, while rare variants (MAF < 0.01) were retained for downstream analysis. Synonymous variants were assumed to be benign or likely benign. Potential functional impact of missense variants was assessed using CADD‐Phred scores. For previously unreported missense variants in the new candidate genes, structural functional effects were further assessed using HOPE and Phyre2. The potential impact of the variants on splicing was evaluated using Alamut Visual Plus software version 1.13 (Interactive Biosoftware, Rouen, France), which employs five different algorithms: SpliceSiteFinder‐like, MaxEntScan, NNSPLICE, GeneSplicer, and HumanSplicingFinder. The following public databases were used for the interpretation of the variants: ClinVar (https://www.ncbi.nlm.nih.gov/clinvar/, accessed 26 November 2021), Human Genome Mutation Database (HGMD, http://www.hgmd.cf.ac.uk/ac/index.php3). The functional impact of SNVs was evaluated using multiple *in silico* prediction tools, including PolyPhen‐2 (Adzhubei et al. 2010), SIFT (Ng 2003), PROVEAN, MutationTaster (Schwarz et al. 2014), and Alamut Visual Plus for assessment of splicing effects. Variant prioritization focused on rare, potentially deleterious variants based on population allele frequency, *in silico* pathogenicity predictions, inheritance patterns, and established gene disease associations.

As part of the interpretation workflow, variants identified within the curated neurodevelopmental disorder (NDD) gene panel were prioritized based on gene‐disease association, inheritance pattern, population allele frequency, predicted functional impact, phenotypic concordance, and evidence from the literature and public disease databases. Variant filtering was performed using Alissa Interpret (Agilent Technologies), applying population allele frequency thresholds from the gnomAD v2.1 non‐neuro database (0.1% for autosomal dominant and X‐linked dominant disorders; 0.5% for autosomal recessive and X‐linked recessive disorders). Trio‐based analyses considered multiple inheritance models, including *de novo* dominant, compound heterozygous recessive, homozygous recessive, X‐linked recessive, inherited dominant, and imprinting‐related mechanisms. Candidate variants were prioritized according to Human Phenotype Ontology (HPO)‐based phenotypic concordance.

The analysis was restricted to variants relevant to ASD and related neurodevelopmental disorders. Incidental findings unrelated to the study phenotype were not systematically evaluated.

Variants were also interpreted and classified according to the ACMG/AMP guidelines (Richards et al. 2015), incorporating ClinGen gene and variant‐specific recommendations when available. Loss‐of‐function variants, including frameshift, stop‐gain, and canonical splice‐site variants, were evaluated under the PVS1 criterion but were not automatically classified as pathogenic. Final variant classification integrated population allele frequency data, *in silico* prediction tools, and evidence from curated variant databases and published literature.

## 3 Results

### 3.1 Clinical Characteristics of Patients With ASD

A total of 34 probands with a clinical diagnosis of ASD and their parents were initially recruited, including 27 males (79.4%) and 7 females (20.6%). Detailed phenotypic information was collected for all participants (Tables 1 and 2). First‐tier genetic testing, including Fragile X screening, identified three positive cases, which were excluded from further analysis. Trio‐based WES was subsequently performed in the remaining 31 patients. ASD severity was classified as mild in 1/31 participants (3.2%), moderate in 8/31 (25.8%), and severe in 22/31 (71.0%).

**TABLE 1:** Likely pathogenic variants.

ID	Age (years)	Sex	Clinical feature	CNV	Included OMIM genes	Gene NM_ & location	Coding change	Protein change	Inheritance & origin	Variant function	SFARI gene score	OMIM morbid phenotype
ASD001C/ID	11	M	Autism, delayed fine motor development, delayed gross motor development, delayed speech and language development and Intellectual disability	20q13.32	GNAS	NC_000020.10:g.[pter_qter];[(?_ 57474991_57475024_?)]del	—	—	Maternal	—	2	Pseudohypoparathyroidism(AD), Osseous heteroplasia, progressive(AD)
ASD004C/AC	10	M	Autism, abnormal, dysmorphic features delayed gross motor development, global developmental delay hypotonia, Intellectual disability, seizure, specific learning disability	1p35.3‐p35.2	PUM1, EPB41, MECR, SDC3	NC_000001.10:g.[pter_qter=];[(?_28733946_32280948_?)]del	—	—	PUM1(AD) EPB41(AD) Denovo	—		Spinocerebellar ataxia 47 (AD), Neurodevelopmental disorder with motor abnormalities, seizures, and facial dysmorphism (AD)
ASD066C/IAA	8	M	Autism, abnormal dysmorphic features, delayed speech and language development, Intellectual disability, seizure	—	GABRB3	NM_000814.6:, Chr15:27017855, Exon; 2	c.145G>A	p.Asp49Asn	AD *De novo*	nonsynonmous	**1**	Developmental and epileptic encephalopathy 43 ((AD), Epilepsy, childhood absence, susceptibility to, 5)
ASD071C/IUE	9	M	Autism, abnormal dysmorphic features, delayed gross motor development, delayed speech and language development, global developmental delay, Intellectual disability, seizure, specific learning disability	—	SYNGAP1	NM_006772.3; Chr6:33414346, intron; 17	c.3583‐6G>A	—	AD *De novo*	—	**1S**	Intellectual developmental disorder (AD)

**TABLE 2:** VUS variants.

ID	Sex	Age	Clinical feature	Location (GRCh37)	Gene NM_	Included OMIM genes	Exon	Coding change	Protein change	variant inheritance	Variant function	SFARI gene score	OMIM morbid phenotype
ASD015C/INB	M	3	Autism, cleft upper lip, delayed gross motor development, delayed speech and language development, global development delay, Intellectual disability, history of oligohydramnios, specific learning disability	Chr22:51160262	NM_001372044.2	SHANK3	22	c.4226C>T	p.Pro1409Leu	AD *De novo*	nonsynonymous	**1S**	Phelan‐McDermid syndrome(AD), Schizophrenia 15(AD)
ASD037C/GB	M	6	Autism, delayed speech and language development, Neonatal seizures, Intellectual disability, Prolonged neonatal jaundice	Chr21:34014214	NM_003895.3	SYNJ1	29	c.3697G>C	p.Ala1233Pro	AR Maternal	nonsynonymous	2	Parkinson disease 20, early‐onset (AR), Developmental and epileptic encephalopathy 53(AR)
				Chr21:34015767	NM_003895.3		27	c.3548G>A	p.Gly1183Asp	AR paternal	nonsynonimous	2	Parkinson disease 20, early‐onset (AR), Developmental and epileptic encephalopathy 53(AR)
ASD035C/NMB	M	5	Autism, Intellectual disability	Chr3:44819652	NM_020242.3	KIF15	4	c.292A>G	p.Met98Val	AR Maternal	nonsynonymous		?Braddock‐Carey syndrome 2(AR)
				Chr3:44872513	NM_020242.3		Intron; 26	c.3171 + 3A>G	p.?	AR paternal	—		?Braddock‐Carey syndrome 2(AR)
UR/2024/ASD047C/HS	M	6	Autism, cataract, delayed speech and language development, Intellectual disability, seizures	ChrX:53566722	NM_031407.7	HUWE1	75	c.11528A>C	p.Glu3843Ala	XL *De novo*	nonsynonymous	**S**	Intellectual developmental disorder, X‐linked syndromic, Turner type(x‐linked)
ASD051C/HNS	M	5	Autism, delayed speech and language development, Delayed gross motor development, Intellectual disability, Specific learning disability	Chr18:43458352	NM_020964.3	EPG5	34	c.5931G>C	p.Glu1977Asp	AR *De novo*	nonsynonymous		Vici syndrome(AR)

The cohort was clinically heterogeneous, with participants ranging in age from 2 to 18 years and presenting with a broad spectrum of neurodevelopmental and neurological features. Intellectual disability was observed in 30/31 participants (96.8%), while delayed speech and language development was reported in 25/31 (80.6%). Learning difficulties and global developmental delay were present in 18/31 (58.1%) and 17/31 (54.8%) individuals, respectively. Delayed gross motor development was observed in 14/31 participants (45.2%), whereas seizures and dysmorphic features were reported in 8/31 (25.8%) and 7/31 (22.6%), respectively. A detailed summary of the clinical features is provided in Table S1.

**FIGURE 2:**
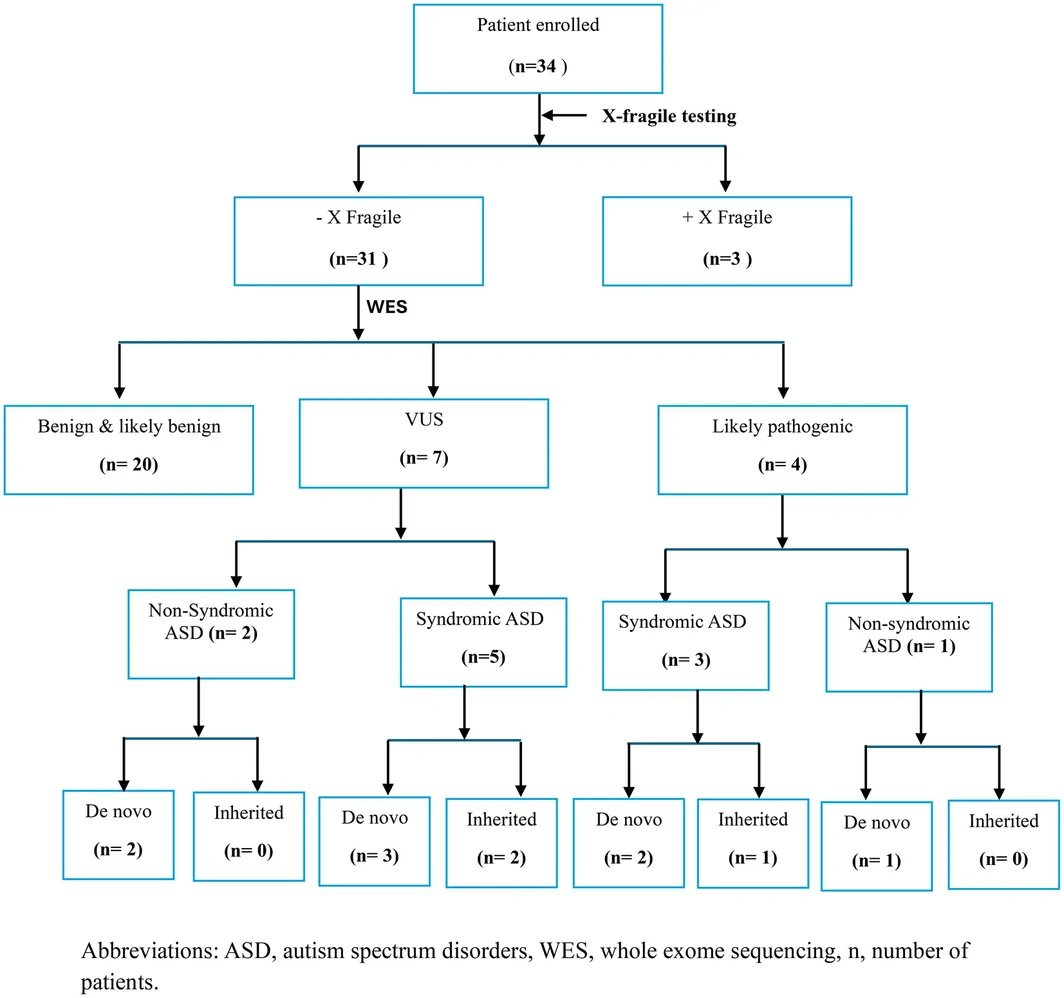
The findings summary for enrolled patients.

Of the four individuals with likely pathogenic variants, three were classified as having syndromic ASD and one as having non‐syndromic ASD. All four individuals harboring likely pathogenic variants were classified as having severe ASD. The individuals with likely pathogenic variants commonly presented with intellectual disability, epilepsy, and/or global developmental delay. Although these findings suggest a higher diagnostic yield among syndromic cases, the modest sample size and limited number of likely pathogenic variants preclude robust statistical assessment of genotype–phenotype associations. These findings are summarized in Figure 2.

### 3.2 Whole Exome Sequencing (WES)

Analysis identified a total of 11 rare genomic findings of potential clinical significance in 9 patients. These comprised four likely pathogenic findings identified in four different patients (12.9%; Table 1), including two single‐nucleotide variants and two copy‐number variants, and seven variants of uncertain significance (VUS) identified in 5 patients (16.1%; Table 2). All remaining variants were classified as benign or likely benign. Among the identified variants, three likely pathogenic variants and five VUS occurred in individuals with syndromic ASD, whereas one likely pathogenic variant and two VUS were identified in individuals with non‐syndromic ASD (Figure 2). The identified variants involved genes associated with synaptic signaling, neuronal development, and cellular homeostasis: *GABRB3*, *SYNGAP1*, *SHANK3*, *HUWE1*, and *SYNJ1*, as well as structural variants involving chromosome 1p35.3‐p35.2 and the *GNAS* locus at 20q13.32. These findings highlight the genetic heterogeneity of ASD and suggest a higher frequency of clinically relevant variants among individuals with syndromic ASD in this cohort.

### 3.3 Copy‐Number Variants (CNVs)

Two likely pathogenic CNVs were identified in two patients (6.4%). Table 1 summarizes the CNVs identified in this study that have sufficient evidence to be classified as likely pathogenic.

Patient 1 (case 38) harbored a *de novo* heterozygous deletion of +/‐3,547Kb at chromosome 1p35.3‐p35.2, encompassing 89 genes and 29 protein‐coding genes. Based on the ACMG/ClinGen interpretation guidelines, this CNV was classified as likely pathogenic.

Patient 2 (case 26) carried a heterozygous 35‐bp deletion at chromosome 20q13.32, located in intron 3 of the *GNAS* gene. This CNV was classified as likely pathogenic in accordance with ACMG and ClinGen guidelines.

### 3.4 Likely Pathogenic Single‐Nucleotide Variants (SNVs)

Two *de novo* heterozygous variants classified as likely pathogenic were identified in *GABRB3* and *SYNGAP1* genes. Both variants were heterozygous and confirmed as *de novo* by trio analysis.

In case 54, a *de novo* missense variant in *GABRB3* (NM_000814.6:c.145G>A, p.[Asp49Asn]) was absent from population databases, including Exome Sequencing Project, 1000 Genomes Project, Exome Aggregation Consortium, and gnomAD. This variant was predicted to be deleterious *in silico* prediction models (REVEL) and is located in the neurotransmitter‐gated ion channel protein domain, a region showing regional missense constraint.

Case 57 harbored a *de novo* splice‐region variant in *SYNGAP1* (NM_006772.3:c.3583‐6G>A p.(?)). Although the precise impact on the protein cannot be predicted, *in silico* splicing analyses (MaxEnt, SSF, GeneSplicer, SpliceAI) consistently predicted exon 16 skipping. In addition, published in vitro functional studies demonstrated disruption of the normal splicing (Li et al. 2024).

### 3.5 Variants of Uncertain Significance (VUS)

Seven variants of uncertain significance (VUS) were identified in *SYNJ1, KIF15, SHANK3, HUWE1*, and *EPG5*. Two genes, *SYNJ1* and *KIF15*, each harbored two heterozygous missense variants.

In case 35, two *SYNJ1* variants were identified: NM_003895.3:c.3697G>C, p.(Ala1233Pro), inherited from the mother, and NM_003895.3:c.3548G>A, p.(Gly1183Asp), inherited from the father. Both variants were absent from control populations, including the Exome Sequencing Project, the 1000 Genomes Project, ExAC, and gnomAD. Based on parental inheritance, the variants are in trans, consistent with a compound heterozygous configuration. In patient case 40, two *KIF15* variants were identified: NM_020242.3:c.292A>G, p.(Met98Val), inherited from the mother, and NM_020242.3:c.3171 + 3A>G, p.(?), inherited from the father. Both variants were absent from control populations and were inherited in trans.

In addition, a *de novo* heterozygous *SHANK3* variant (NM_001372044.2:c.4226C>T, p.Pro1409Leu) was identified in case 31. This variant, located in exon 22, was absent from the Exome Sequencing Project, the 1000 Genomes Project, ExAC, and gnomAD, and represents a novel variant.

In case 41, a heterozygous variant in *HUWE1* (NM_031407.7:c.11528A>C) was identified. Although the inheritance pattern raises the possibility of compound heterozygosity in other genes, the available evidence is insufficient to establish pathogenicity. In case 42, a heterozygous variant in *EPG5* (NM_020964.3:c.5931G>C, p.Glu1977Asp) was identified. This variant is located within a region with high missense constraint and was absent from population databases. Although *EPG5* is associated with the autosomal recessive Vici syndrome (OMIM *615068; #242840), the available evidence is insufficient to support pathogenic classification of this variant.

## 4 Discussion

This study represents one of the first comprehensive evaluations of rare genetic variation in pediatric autism spectrum disorder (ASD) in a Rwandan cohort using trio‐based whole‐exome sequencing (WES). Based on likely pathogenic variants, the diagnostic yield was 12.9%, increasing to 29.03% when including variants of uncertain significance (VUS) with strong phenotypic concordance also considered. These findings demonstrate the clinical utility of WES in this cohort. The diagnostic yield observed in this study is comparable to that reported in other trio‐based WES studies of ASD and related neurodevelopmental disorders, which typically range between approximately 10% and 30%, depending on cohort characteristics and inclusion criteria (Fu et al. 2022; Satterstrom et al. 2020). However, given the modest sample size and limited representation of African populations in genomic studies, larger cohorts are needed before broader conclusions regarding the genetic architecture of ASD in African populations can be drawn.

Notably, three of the four individuals harboring likely pathogenic variants were classified as having syndromic ASD and presented with features such as intellectual disability, global developmental delay, epilepsy, and dysmorphic features. This observation is consistent with previous studies reporting higher diagnostic yields among individuals with ASD accompanied by additional neurodevelopmental manifestations (Satterstrom et al. 2020; Arora et al. 2025), although the modest sample size precludes definitive genotype–phenotype conclusions. The identified variants predominantly affected genes involved in synaptic signaling, neuronal development, and intracellular homeostasis, supporting the growing evidence that disruption of these pathways contributes to ASD pathogenesis.

Rare copy‐number variants (CNVs) are well‐established contributors to ASD risk, with affected individuals exhibiting a higher burden of rare CNVs than neurotypical controls (Satterstrom et al. 2020). Several recurrent ASD‐associated CNV hotspots have been described, including 16p11.2 and 15q11‐q13 (Szafranski et al. 2010; Moreira et al. 2014), and recently systematic evaluations have identified numerous additional candidate regions associated with neurodevelopmental disorders (Tamada and Takumi 2025). Two of four likely pathogenic findings identified in this cohort were CNVs, including a *de novo* 1p35.3‐p35.2 deletion and a deletion involving the *GNAS* locus at 20q13.32. The large *de novo* 1p35.3‐p35.2 microdeletion identified in case 38 was associated with autism, hypotonia, seizures, and developmental delay. This phenotype is consistent with the combined effects of multiple dosage‐sensitive genes within the deleted region, including *PUM1, SDC3*, and *MECR*, which are implicated in neurodevelopment and motor function (Heimer et al. 2016; Gennarino et al. 2018). The multisystemic presentation likely reflects the cumulative impact of contiguous gene loss, a well‐established mechanism in genomic disorders (Lupski 2015). Although deletions involving this region have rarely been reported in individuals with ASD, the clinical presentation observed in this patient supports the pathogenicity of the CNV and suggests that haploinsufficiency within this interval may contribute to syndromic neurodevelopmental disorders.

In case 26, a deletion involving the GNAS locus at 20q13.32 was identified in a patient with intellectual disability and developmental delay. Although inherited from an unaffected parent, the patient's clinical features are consistent with the known role of GNAS in neurodevelopment (Y. Liu et al. 2022), highlighting the complexity of interpreting inherited variants in ASD and the potential role of variable penetrance and expressivity.

Among single‐nucleotide variants, the identification of *de novo* variants in GABRB3 (case 54) and *SYNGAP1* (case 57) further supports the role of synaptic dysfunction in ASD. In case 54, the presence of early‐onset seizures, intellectual disability, and language delay is consistent with developmental and epileptic encephalopathy associated with *GABRB3*. The *de novo* variant identified in this gene is predicted to impair GABAergic inhibitory signaling (Pizzarelli and Cherubini 2011), which may contribute to the combined epilepsy and ASD phenotype observed in this patient. Similarly, the clinical presentation in case 57, including global developmental delay, intellectual disability, and dysmorphic features, aligns with *SYNGAP1*‐related disorders (Xie et al. 2025), which are characterized by impaired synaptic plasticity, altered neuronal excitability, and disrupted neurodevelopment (Llamosas et al. 2021). Together, these findings reinforce the contribution of synaptopathies to ASD pathogenesis.

Several variants of uncertain significance (VUS) showed notable genotype–phenotype concordance. Altered synapse development and signaling resulting from *SHANK3* dysfunction are well‐established contributors to ASD pathogenesis (Durand et al. 2007). In case 31, a *de novo* variant in *SHANK3*, a well‐established ASD gene, was identified in a patient presenting with autism, global developmental delay, and craniofacial anomalies, features consistent with the clinical spectrum of Phelan‐McDermid syndrome (Durand et al. 2007; Phelan and McDermid 2011). Although this variant is currently classified as a VUS, its phenotypic overlap with previously reported *SHANK3*‐associated disorders suggests potential clinical relevance. However, additional genetic and functional evidence is required before its contribution to the observed phenotype can be established.

In case 35, compound heterozygous variants in *SYNJ1* were identified in a patient presenting with epilepsy, intellectual disability, and metabolic abnormalities, features that overlap with previously reported *SYNJ1*‐associated phenotypes (Samanta and Arya 2020; Maj et al. 2023). This phenotypic overlap is consistent with the established role of *SYNJ1* in synaptic vesicle recycling and neurodevelopment (Drouet and Lesage 2014). Similarly, in case 40, variants in *KIF15* were identified in a patient with autism and intellectual disability, consistent with its proposed role in neuronal development, although its association with ASD remains to be established (Liu et al. 2025). In case 41, a variant in *HUWE1* was identified in a patient presenting with autism, seizures, and cataracts. Given previous reports implicating *HUWE1* in syndromic neurodevelopmental disorders (De Falco et al. 2025), this finding represents a plausible candidate for further investigation. In case 42, a variant in *EPG5* was identified in a patient with global developmental delay and neurodevelopmental impairment. Although *EPG5* has not been widely implicated in ASD, its established role in multisystemic disorders affecting cellular homeostasis (Dafsari et al. 2024), together with emerging evidence linking autophagy and intracellular trafficking to neurodevelopmental disorders (Frickel et al. 2025), suggests that this variant warrants further investigation. While these variants remain classified as VUS, their phenotypic overlap with previously reported disorders suggests potential clinical relevance; however, additional genetic and functional evidence is required before any contribution to ASD can be established.

Overall, the likely pathogenic variants identified in this study implicate biological pathways related to synaptic signaling and neuronal development, consistent with current models of ASD pathogenesis. Several additional variants of uncertain significance were identified in genes involved in synaptic function, neuronal development, intracellular trafficking, and cellular homeostasis. However, their contribution to ASD remains uncertain and requires further validation through functional studies and the identification of additional affected individuals.

The findings of this study are consistent with current models of ASD genetics, which support contributions from both *de novo* and inherited rare variants. Recent large‐scale sequencing studies have shown that *de novo* protein‐altering variants are enriched in individuals with ASD, particularly those with intellectual disability, developmental delay, and other syndromic features, whereas inherited rare variants also contribute substantially to ASD risk through diverse biological pathways (Satterstrom et al. 2020; Fu et al. 2022). In our cohort, the identification of *de novo* variants in established ASD‐associated genes, including GABRB3 and SYNGAP1, is consistent with these observations, while inherited variants in other neurodevelopmental genes further illustrate the genetic heterogeneity of ASD. Together, these findings support the contribution of multiple classes of rare genetic variation to ASD through convergent neurodevelopmental and synaptic pathways.

Importantly, this study expands genomic data from an underrepresented African population and represents one of the few trio‐based WES studies of ASD conducted in sub‐Saharan Africa. Studies of ASD genetics in African populations are scarce, and trio‐based WES has rarely been applied; consequently, diagnostic yields in these populations are not yet well established (Frickel et al. 2025; Hakizimana et al. 2024). Despite harboring the greatest human genetic diversity, African populations remain markedly underrepresented in global genomic reference databases (Martin et al. 2018; Peterson et al. 2019). This underrepresentation poses significant challenges for variant interpretation and likely contributes to the relatively high proportion of VUS identified in this study. Expanding genomic research in African populations is therefore essential to improve variant classification, enhance diagnostic accuracy, and promote equitable implementation of genomic medicine (Caswell‐Jin et al. 2018; Popejoy et al. 2018). In this context, trio‐based WES represents a valuable diagnostic approach that can reduce the diagnostic odyssey, inform clinical management, and provide recurrence risk information for affected families.

This study has several limitations. First, this was a single‐site study with a modest sample size, which may limit statistical power and the generalizability of the findings. Consequently, the observed enrichment of clinically relevant likely pathogenic variants among individuals with syndromic ASD should be interpreted as a descriptive finding. Second, WES does not capture all classes of genetic variation, including non‐coding regulatory variants and some structural variants, and environmental and epigenetic contributors to ASD were not assessed. Finally, variant filtering and interpretation relied on publicly available genomic reference databases, including gnomAD, ClinVar, DGV, DECIPHER, and OMIM, which incompletely represent African populations. Consequently, allele frequency estimates and the interpretation of rare variants may be less accurate in the Rwandan population, underscoring the need for expanded African genomic reference datasets.

## 5 Conclusions

This study expands understanding of the genetic architecture of ASD in an underrepresented Rwandan pediatric cohort by identifying likely pathogenic variants and variants of uncertain significance through trio‐based whole‐exome sequencing. The findings highlight the genetic heterogeneity of ASD and suggest a higher frequency of likely pathogenic variants among individuals with syndromic ASD. Importantly, this study contributes genomic data from an underrepresented African population and supports the value of trio‐based WES for the genetic evaluation of ASD. Continued inclusion of ancestrally diverse populations, together with functional validation through transcriptomic analyses (RNA sequencing), splicing assays for variants predicted to affect RNA processing, and patient‐derived cellular or induced pluripotent stem cell (iPSC)‐based neuronal models, as well as longitudinal phenotyping, will be essential to improve variant interpretation, advance understanding of ASD biology, and promote the equitable implementation of genomic medicine.

## Author Contributions

**Olivier Hakizimana:** investigation, methodology, validation, visualization, writing – original draft, software, formal analysis, project administration. **Janvier Hitayezu:** project administration, supervision, investigation, methodology, validation, writing – review and editing. **Jeanne P. Uyisenga:** investigation, writing – review and editing, methodology, formal analysis, supervision. **Norbert Dukuze:** methodology, investigation, project administration. **Marie Viviane Akimana:** investigation, methodology, project administration. **Laurence Mizero:** investigation, methodology, project administration. **Claudine Bampire:** investigation, methodology, data curation. **Charles Mudenge:** investigation, methodology, supervision, validation. **Xavier Kanyambari Butoto:** methodology, validation, investigation, supervision. **Laura Helou:** validation, methodology, data curation, software, formal analysis. **Benoit Charloteaux:** methodology, validation, software, formal analysis, data curation. **Jean‐Hubert Caberg:** methodology, validation, visualization, software, formal analysis, data curation. **Vinciane Dideberg:** methodology, validation, visualization, software, formal analysis, data curation. **Leonor Palmeira:** methodology, validation, visualization, supervision, data curation, software, formal analysis. **Abdullateef Isiaka Alagbonsi:** conceptualization, investigation, writing – review and editing, methodology, validation, visualization, software, formal analysis, data curation, supervision. **Vincent Bours:** investigation, methodology, validation, visualization, writing – review and editing, software, formal analysis, supervision. **Annette Uwineza:** conceptualization, investigation, funding acquisition, methodology, validation, visualization, writing – review and editing, project administration, supervision, data curation, software, resources.

## Funding

This study was sponsored by the African Academy of Sciences through the African Research Initiative for Scientific Excellence (ARISE), pilot programme (grant number ARISE‐PP‐40).

## Ethics Statement

Ethics considerations were central to this study involving children diagnosed with ASD and their parents. The study received full approval from the University of Rwanda Institutional Review Board (No 298/CMHS IRB/2023) and the Ndera Neuropsychiatric Teaching Hospital Ethics Committee (N^o^ 045/NNPTHEC/2023).

## Consent

Informed consent was obtained from all participants after explaining the study's objectives, procedures, and their right to withdraw at any time. Confidentiality and privacy were strictly maintained, with data anonymized and access restricted to authorized research team members. All procedures adhered to established ethics guidelines, ensuring participants' rights, dignity, and well‐being were fully protected.

## Conflicts of Interest

The authors declare no conflicts of interest.

## Supporting information


**Table S1:** Clinical characteristics of children with autism spectrum disorder included in the whole‐exome sequencing (WES) cohort (*N* = 31).

## Data Availability

The datasets used and/or analyzed during the current study are available from the corresponding author on reasonable request.
